# Presentation and management of *N*-acetylglutamate synthase deficiency: a review of the literature

**DOI:** 10.1186/s13023-020-01560-z

**Published:** 2020-10-09

**Authors:** Aileen Kenneson, Rani H. Singh

**Affiliations:** 1 Department of Human Genetics, Emory University, Atlanta, GA USA; 2 Department of Pediatrics, Emory University, Atlanta, GA USA

**Keywords:** *N*-Acetylglutamate synthase, NAGS, *N*-Acetylglutamate synthase deficiency, Inherited metabolic disorder, Urea cycle, Hyperammonemia, Carbamylglutamate, Carbaglu

## Abstract

**Background:**

*N*-Acetylglutamate synthase (NAGS) deficiency is an extremely rare autosomal recessive metabolic disorder affecting the urea cycle, leading to episodes of hyperammonemia which can cause significant morbidity and mortality. Since its recognition in 1981, NAGS deficiency has been treated with carbamylglutamate with or without other measures (nutritional, ammonia scavengers, dialytic, etc.). We conducted a systematic literature review of NAGS deficiency to summarize current knowledge around presentation and management.

**Methods:**

Case reports and case series were identified using the Medline database, as well as references from other articles and a general internet search. Clinical data related to presentation and management were abstracted by two reviewers.

**Results:**

In total, 98 cases of NAGS deficiency from 79 families, in 48 articles or abstracts were identified. Of these, 1 was diagnosed prenatally, 57 were neonatal cases, 34 were post-neonatal, and 6 did not specify age at presentation or were asymptomatic at diagnosis. Twenty-one cases had relevant family history. We summarize triggers of hyperammonemic episodes, diagnosis, clinical signs and symptoms, and management strategies. DNA testing is the preferred method of diagnosis, although therapeutic trials to assess response of ammonia levels to carbamylglutamate may also be helpful. Management usually consists of treatment with carbamylglutamate, although the reported maintenance dose varied across case reports. Protein restriction was sometimes used in conjunction with carbamylglutamate. Supplementation with citrulline, arginine, and sodium benzoate also were reported.

**Conclusions:**

Presentation of NAGS deficiency varies by age and symptoms. In addition, both diagnosis and management have evolved over time and vary across clinics. Prompt recognition and appropriate treatment of NAGS deficiency with carbamylglutamate may improve outcomes of affected individuals. Further research is needed to assess the roles of protein restriction and supplements in the treatment of NAGS deficiency, especially during times of illness or lack of access to carbamylglutamate.

## Background

**Fig. 1:**
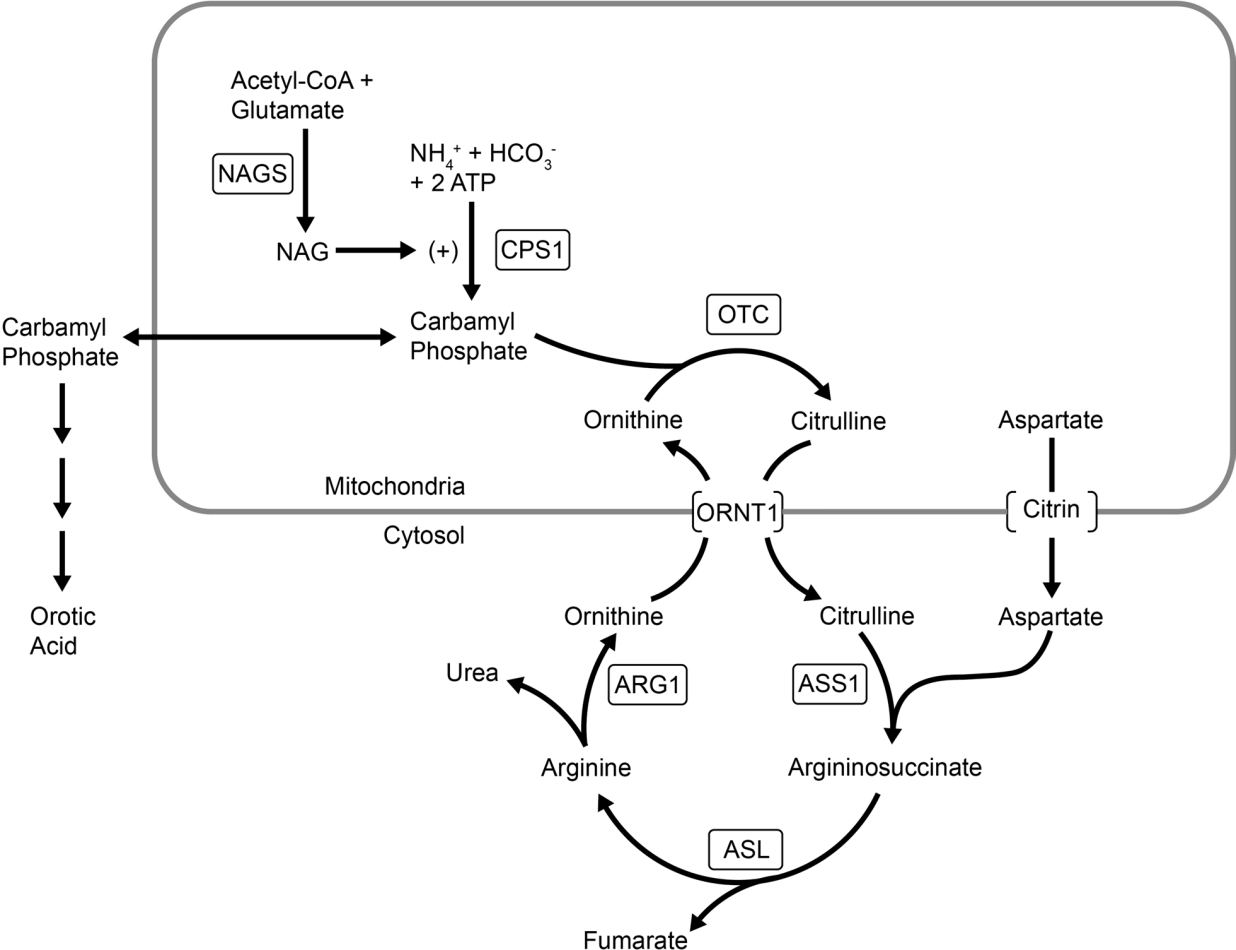
The urea cycle. Adapted from https://www.genereviews.org/ © 1993–2020 University of Washington [1]. *ARG1* arginase, *ASL* argininosuccinic acid lyase, *ASS1* argininosuccinic acid synthetase, *CPS1* carbamyl phosphate synthetase 1, *NAG* *N*-acetylglutamate, *NAGS* *N*-acetylglutamate synthase, *ORNT1* ornithine translocase, *OTC* ornithine transcarbamylase

Ammonia is the toxic product of excess nitrogen in the body. Ammonia is detoxified in the liver by conversion to urea via a series of enzymatic reactions in the urea cycle (Fig. 1) [1]. Disorders of the urea cycle result in episodes of hyperammonemia, which can present within the first few days of life in patients with more severe enzyme deficiency or precipitated later in life by an event such as an illness in patients with milder enzyme deficiencies [2]. Hyperammonemia can lead to coma, brain damage, and death. Conventional treatment of hyperammonemia associated with urea cycle defects includes ammonia scavengers (such as sodium benzoate and sodium phenylacetate), arginine supplementation to maximize urea cycle function, and hemodiafiltration [2].

The rarest of the urea cycle disorders is *N*-acetylglutamate synthase (NAGS) deficiency, an autosomal recessive disorder first described in 1981 [3], with an estimated incidence of less than one in 2,000,000 livebirths [4]. NAGS is a mitochondrial enzyme that catalyzes the formation of *N*-acetylglutamate (NAG) from glutamate and acetyl coenzyme-A [5] in a reaction that is stimulated by arginine [6, 7]. NAG is an essential activator of carbamyl phosphate synthetase 1 (CPS1), the first enzyme of the urea cycle in mammals [8, 9]. Because NAG is an essential activator of CPS1, deficiency in either NAGS or CPS1 leads to a similar biochemical phenotype, including elevated glutamine, reduced citrulline, normal orotic acid, and elevated ammonia. NAGS deficiency and CPS1 deficiency can be distinguished by molecular genetic testing [2]. It is important to obtain an accurate diagnosis because NAGS deficiency can be successfully managed with carbamylglutamate, an orally bioavailable aminoacylase-resistant NAG analog that activates CPS1 [9, 10].

We conducted a systematic literature review of cases of NAGS deficiency to summarize current knowledge of the presentation and management of this rare disease.

## Main text

### Literature search

A search of the Medline database using the search terms “NAGS” or “*N*-acetylglutamate synthase” was conducted in April 2020, identifying 222 articles. Articles that were not in English, not focused on human subjects, or not involving one or more cases of NAGS deficiency were excluded. This resulted in 38 articles. The reference lists on these articles were searched and led to the identification of seven additional relevant articles or abstracts. A further three articles or abstracts were identified using a general internet search. Several patient cases were presented in more than one article and this information was consolidated when identified. Two reviewers independently extracted data from each article using a form. Extracted data from the two reviewers were compared and discrepancies were resolved. Data extracted for each case included: sex, age at presentation, suspected trigger, presenting symptoms, biomarker levels at presentation (including laboratory reference ranges), diagnostic test results (liver NAGS enzyme assay and DNA testing), acute treatment, long-term treatment, and remaining symptoms. Biomarker data included plasma ammonia (at presentation and at peak), plasma amino acids (glutamine, alanine, arginine, and citrulline), urine orotic acid, and other abnormal test results. In total, 98 cases of NAGS deficiency from 79 families in 48 articles or abstracts were identified. A summary of these cases is available in Additional file 1: Table S1.

#### Presentation of NAGS deficiency

Of the 98 cases of NAGS deficiency identified in the literature, 1 was diagnosed prenatally, 57 were neonatal cases (birth to age 28 days), 29 were post-neonatal, 5 were described as late-onset but did not specify age at presentation, 5 were asymptomatic at diagnosis, and 1 did not specify age at onset. The oldest age at presentation was 59 years. Consanguinity was reported in 21 cases from 14 families. Relevant family history was reported in 21 cases, including family history of death associated with hyperammonemia (n = 5), hyperammonemia (n = 3), NAGS deficiency (n = 4), infant or early childhood death (n = 1), sibling death at unspecified age (n = 1), carbamylglutamate-responsive hyperammonemia (n = 1), and relevant family history without specific details reported (n = 4).

Approximately two-thirds of cases included details about clinical signs and symptoms at presentation. Signs and symptoms that were present in two or more cases are presented in Table 1. Neonatal cases often presented with poor feeding or feeding intolerance, vomiting, lethargy, hypertonia and/or hypotonia, seizures, and tachypnea. Common presenting symptoms in later onset cases included vomiting, confusion or disorientation, ataxia, lethargy, decreased level of consciousness, seizures, and hypotonia. In some cases of later onset NAGS deficiency, there was a history of avoidance of high-protein foods [11–14]. In addition to elevated ammonia, the most commonly-reported biomarkers in blood were elevated glutamine and alanine, decreased citrulline, and respiratory alkalosis. Urine orotic acid was normal in 26 cases and decreased in seven cases.

**Table 1:** Presenting signs and symptoms of NAGS deficiency described in the literature

Neonatal presentation—clinical symptoms^a^	Post-neonatal presentation—clinical symptoms^a^	Biochemical signs in blood^a^
n = 38	n = 28	
Poor feeding/anorexia/intolerance (n = 16)	Vomiting (n = 12)	Elevated ammonia (n = 43)
Vomiting (n = 13)	Confusion/disorientation (n = 7)	Elevated glutamine (n = 29)
Coma (n = 12)	Ataxia (n = 7)	Decreased citrulline (n = 20)
Lethargy (n = 12)	Lethargy (n = 7)	Elevated alanine (n = 9)
Hypotonia (n = 7)	Avoidance of high-protein	Decreased arginine (n = 7)
Hypertonia (n = 6)	Foods (n = 6)	Respiratory alkalosis (n = 6)
Seizures (n = 6)	Coma (n = 6)	Decreased isoleucine (n = 4)
Tachypnea (n = 6)	Decreased level of	Elevated lactate (n = 4)
Encephalopathy (n = 4)	Consciousness (n = 6)	Decreased leucine (n = 3)
Respiratory distress (n = 4)	Seizures (n = 5)	Decreased ornithine (n = 3)
Unresponsiveness (n = 4)	Hypotonia (n = 5)	Elevated arginine (n = 3)
Abnormal movements (n = 3)	Small head circumference (n = 5)	Elevated lysine (n = 3)
Hepatomegaly (n = 3)	Abnormal reflexes (n = 4)	Coagulation problems (n = 2)
Poor sucking (n = 3)	Weight at low percentile (n = 4)	Decreased BUN (n = 2)
Somnolence/drowsiness (n = 3)	Combativeness/aggression (n = 3)	Elevated AST (n = 2)
Convulsions (n = 2)	Hepatomegaly (n = 3)	Elevated phenylalanine (n = 2)
Hyperthermia (n = 2)	Nausea (n = 3)	Hypoglycemia (n = 2)
Irritability (n = 2)	Somnolence/drowsiness (n = 3)	
Tremor/trembling (n = 2)	Anorexia (n = 2)	
	Bizarre behavior (n = 2)	
	Convulsions (n = 2)	
	Headaches (n = 2)	
	Involuntary movements (n = 2)	
	Restlessness (n = 2)	
	Poor growth (n = 2)	

^a^Clinical signs or symptoms reported in two or more cases in the literature

Suspected triggers in post-neonatal cases that were reported in the literature included febrile illness [14–16], pregnancy and delivery [17–19], a high-protein meal [11, 20], the introduction of cow milk [21], low caloric intake and dehydration [13], an upper respiratory illness [22], a vehicle accident [23], and a fractured pelvis [24].

#### Diagnosis of NAGS deficiency

Liver NAGS enzyme activity was used in the diagnosis of NAGS deficiency in 32 of the cases, with details provided in 25 of the cases (Table 2). In cases with no detectable enzyme activity, clinical presentation was within the first week of life [3, 25–28]. Otherwise, there was no correlation between liver NAGS enzyme activity level and age at onset of NAGS deficiency. In neonatal cases, the enzyme activity level varied from 0 to 100% of normal levels without arginine stimulation (n = 12, mean = 37%) and 2–72% when assayed with arginine stimulation (n = 9, mean = 28%). Similar results were observed for post-neonatal onset (without arginine, n = 12, range 0–100%, mean = 40%; with arginine, n = 6, range 4–100%, mean = 40%). Furthermore, decreased NAGS activity was not necessarily indicative of NAGS deficiency, as levels were also decreased in carriers (without arginine, n = 3, range 24–35%, mean = 31%; with arginine, n = 3, range 8–43%, mean = 24%), and were decreased in one case of portosystemic shunt that was tentatively diagnosed as partial NAGS deficiency (47% enzyme activity) until it was found that the patient’s ammonia levels did not respond to carbamylglutamate [29].

**Table 2:** Liver NAGS enzyme activity in NAGS deficiency cases and carriers

References	Onset	NAGS activity without arginine^a^	NAGS activity with arginine^b^	Confirmed with DNA
*Cases*				
[3]	Neonatal	0%	Not reported	
[68] (Pt 7)	Neonatal	15% (not given in U^c^)	Not stimulated	
[68] (Pt 8)	Neonatal	10% (not given in U)	Not stimulated	
[68] (Pt 9)	Neonatal	34% (not given in U)	Stimulated	
[25]	Neonatal	0%	2% 0.44 nmol/min/g protein (Refs. [22–52])	
[69] (Fam 1)	Neonatal	10U = 29%	20U = 14%	√
[70] (Proband)	Neonatal	141U = 100%	74U = 51%	√
[35] (Pt TD)	Neonatal	78U = 100%	104U = 72%	
[35] (Pt YD)	Neonatal	98U = 100%	64U = 44%	√
[26]	Neonatal	< 10% not calculable	Not stimulated	
[27] (Fam 1)	Neonatal	6U = 18%	14U = 10%	√
[27] (Fam 3)	Neonatal	14U = 41%	9U = 6%	
[27] (Fam 6)	Neonatal	0U = 0%	72U = 50%	√
[36]	Neonatal	2.1 nmol/min/g protein (Ref. not given)	4% 2.5 nmol/min/g protein (control:62.8)	
[28] (Case 1)	5 weeks	0% not detectable	Not reported	
[38]	5 months	Not reported	55U = 38%	
[15] (Patient IV-5)	13 months	Not reported	47.9U = 33%	
[14]	3 years	94U = 100%	197U = 100%	√
[12]	4 years	9U = 26%	Stimulated (below ref.)	
[22]	4 years	27% 110 nmol/min/g protein (control: 1160 ± 750)	Not reported	
[11]	12 years	22U = 65%	22U = 15%	√
[20]	12 years	30U = 88%	46U = 32%	
[16]	20 years	14.3U = 42%	21.4U = 15%	√
[18] (No 11)	40 years	5% (not given in U)	4% (not given in U)	√
[18] (No 18)	Late	50% (not given in U)	75% (not given in U)	√
*Carriers*				
[35] (Pt AD)	N/A	12U = 35%	62U = 43%	√
[71] (Pt 2)	N/A	12U = 35%	12U = 8%	√
[71] (Pt 3)	N/A	8U = 24%	32U = 22%	√

^a^Reference > 34 unless otherwise stated

^b^Reference > 144 unless otherwise stated

^c^U = nmol/min/g protein

Results of DNA testing were reported for 76 cases in the literature, with the identification of 47 different pathogenic mutations, although a second mutation was not identified in three of these cases. Most of the mutations were missense, although nonsense, insertion, deletion, splice-site, and enhancer region mutations were also reported. Guffon and colleagues suggested that a therapeutic trial to assess the response of ammonia levels to carbamylglutamate may be helpful in establishing a diagnosis of NAGS deficiency. In their suggested therapeutic trial, carbamylglutamate (200 mg/kg) is administered while other treatments are withheld and ammonia levels are monitored every 2 h for 6 h [21]. However, others have argued that other treatments should not be withheld, and that a combined approach of conventional treatment (including ammonia scavengers, restricted protein diet, and hemodiafiltration as appropriate [2]) and carbamylglutamate should be considered [30, 31].

The recommended initial dose of carbamylglutamate by the manufacturer for treatment of children and adults with acute hyperammonemia is 100–250 mg/kg/day [32]. In the 2019 guidelines for urea cycle disorders, Häberle and colleagues suggested that undiagnosed hyperammonemia should be treated with a 100 mg/kg bolus per NG tube then 25–62.5 mg/kg every 6 h [2]. There was one report of symptoms of intoxication at a dose of 650 mg/kg/day during acute treatment [33].

Diagnosis was also aided in one case report by the use of liquid chromatography tandem mass spectroscopy to identify a reduced NAG level compared to controls [19]. More research is needed to determine if this approach will be useful in diagnosis of NAGS deficiency.

#### Management of NAGS deficiency

The recommended maintenance dose of carbamylglutamate is 10–100 mg/kg/day [32]. In published reports, the prescribed maintenance dose ranged from 15 to 320 mg/kg/day (Table 3). Papers that reported carbamylglutamate use but did not provide dose were not included in this table. The lowest reported asymptomatic dose in the literature was 10 mg/kg/day in a well patient [34]. There were three reports of hyperammonemia that occurred while the patient was receiving carbamylglutamate. The doses associated with hyperammonemia were 10 mg/kg/day (well patient, no protein restriction) [35], 20 mg/kg/day (triggered by illness) [34], and 100 mg/kg/day (triggered by illness) [36].

**Table 3:** Literature review of nutrition management in conjunction with carbamylglutamate treatment

Year	Refs.	Onset	Carbamyl-glutamate mg/kg/day	Protein g/kg/day	Citrulline mg/kg/day	Arginine mg/kg/day	Sodium benzoate mg/kg/day	Outcome
1985	[72] See also [3, 33, 73]	N	180–320	1.5	430^b^	700^b^		Deceased at 9 years
1995	[26]	N	80–100					Normal growth at 1 year
1996	[74] See also [37]	5 months	√ (4 × 200 mg/day)	3.5				Not stated
1997	[16]	20 years	60			50		Gross cerebral dysfunction; paraplegia
1998	[36]	N	100	2.5			70	Normal at 20 months
1998	[11] See also [37]	12 years	100	1.2				Normal at age 24 years
1999	[12]	4 years	100	0.9–1.0				Cognitive impairment
2002	[70]	N	150		√			Developmental delay
2003	[20]	12 years	15					Not stated
2003	[27] See also [35]	N	15–200					Normal at 13 years
2007	[30]	N	50	2.0–3.5^a^				Normal at 2.5 years
2011	[75]	N	√ (200–800 mg/d)					Normal at 20 years
2013	[40]	38 years	150					Short-term memory loss
2014	[34]	N	30					Normal at 3 years
2014	[31]	N	100					Normal at 9 months
2015	[76]	N	75–100	2.0				Poor growth, mild MR, ADHD at 9 years
2016	[77]	N	200					Not stated
2016	[78]	N	100	2.0–2.5				Deceased at 4.5 months
2017	[45]	N	40					Normal at 7 months
2017	[24]	59 years	√ (2 × 600 mg/d)	0.8				Normal
2018	[13]	52 years	24					Discontinuation of symptoms
2018	[14] (Case 1)	3 years	√ (5 × 200 mg/d)	1.0				Not stated
2018	[14] (Case 2)	6 years	50	1.8				Normal growth and development
2018	[14] (Case 3)	6 years	50					Not stated
2020	[44] (Case 1)	N	50	3.5				Normal growth and development at 6 years
2020	[44] (Case 2)	N	50	3.5				Normal growth and development at 4 years
2020	[44] (Case 3)	N	50					Normal growth and development at 16 months

*N* neonatal onset

√, dose not given in mg/kg/day

^a^Ataxia developed when protein was increased to 3.5 g/kg/day

^b^First arginine, then changed to citrulline

Some groups reported that carbamylglutamate treatment was augmented with protein restriction (Table 3). The amount of protein prescribed ranged from 0.8 to 3.5 g/kg/day. In one case, the patient developed ataxia when the protein content was increased to 3.5 g/kg/day while on a maintenance carbamylglutamate dose of 50 mg/kg/day [30]. Some cases published in the 1990s and early 2000s also reported concomitant use of citrulline, arginine, or sodium benzoate, although more recent papers tend to rely solely on carbamylglutamate, with or without protein restriction (Table 3).

Although carbamylglutamate is the standard of care for treatment of NAGS deficiency, a few groups reported long-term nutrition management of NAGS deficiency without carbamylglutamate. Treatment included ammonia scavengers [22, 23, 28, 37, 38], along with protein restriction [23, 28, 37, 38]. Arginine [38] and citrulline [23, 37] supplementation also were provided in some cases to maximize function of the urea cycle. Outcome in cases treated without carbamylglutamate ranged from normal development [28, 37] to profound cognitive impairment [38].

There was a report of two individuals with NAGS deficiency who have been treated by liver transplantation [39]. The first patient with a liver transplant at age 8 months was reported to have global neurodevelopmental delays at 20 months of age. A younger sibling received a liver transplant at age 6 weeks, and at 3 months was reported to have non-oral food intake, but otherwise normal development.

Finally, one patient with late onset recurrent hyperammonemia was treated with oral contraceptives, followed by hysterectomy. This individual was later diagnosed with NAGS deficiency [17, 18].

## Discussion

NAGS deficiency is an extremely rare, autosomal recessive genetic disorder that if left untreated may result in ammonia accumulation and significant morbidity and mortality. NAGS deficiency was first recognized in 1981, and the authors of this literature search identified published information on 97 cases since that time. Presentation of NAGS deficiency varies both by age at onset and symptoms. In addition, both diagnosis and management are not consistent and vary across clinics.

Of the cases that we identified in the literature, two-thirds presented in the neonatal period. The most common presenting symptoms in neonatal cases were poor feeding or feeding intolerance, vomiting, lethargy, hypertonia and/or hypotonia, seizures, and tachypnea. Consistent with the role of NAG in the urea cycle, the most commonly-reported biomarkers in blood, other than elevated ammonia, were elevated glutamine and decreased citrulline. Urine orotic acid levels were not elevated. Respiratory alkalosis was also reported in some cases.

In later onset cases, vomiting, behavioral changes, ataxia, lethargy, decreased level of consciousness, seizures, and hypotonia were common. A variety of physical stressors have been implicated as triggers in post-neonatal cases, and the symptoms can be episodic, especially if the patient normalizes ammonia levels by self-restricting protein intake [2, 11, 13]. Given that the presentation is non-specific, ammonia level assessment in individuals with depressed consciousness or unexplained encephalopathy has been suggested [20, 40].

Consanguinity was reported in 18% of the families included in this report. Consanguinity is associated with significantly increased rates of inherited metabolic disorders in certain communities, such as families of Pakistani, Turkish, Afghan, or Arab origin [41]. Premarital and preconceptional counseling are warranted for consanguineous couples [42, 43].

Diagnosis of NAGS deficiency has evolved over time, from assessment of liver enzyme function to DNA analysis. Enzyme function analysis appears to have limited specificity and sensitivity. Cases with no detectable enzyme activity presented within the first week of life, but among those with residual enzyme function, there was no correlation between enzyme activity and age of onset. Furthermore, several DNA-confirmed cases have been reported to have 100% enzyme activity, one of which also reported 100% activity during arginine stimulation. On the other hand, enzyme activity has been shown to be reduced in some carriers. Because enzyme activity level analysis requires liver biopsy, and can be misleading, DNA testing is preferred for diagnosis. Genetic testing also helps to distinguish between NAGS deficiency and CPS1 deficiency, both of which present with elevated glutamine, reduced citrulline, and normal orotic acid [2]. The large number of associated mutations limits genotype–phenotype correlation in NAGS deficiency, although Sancho-Vaello and colleagues have suggested that phenotype may be dependent on the domain of the gene in which the mutation is located, with missense mutations in the C-terminal domain resembling GNAT-type acetyltransferases more likely to result in neonatal onset cases and missense mutations in the Amino Acid Kinase (AAK) domain more likely to result in later onset cases [37]. Mutations in regulatory regions also have been reported, so it is important to include them in molecular testing. Identification of mutations in the family allows for carrier testing as well as prenatal diagnosis in future pregnancies, leading to prompt administration of carbamylglutamate and prevention of hyperammonemic episodes [44].

A therapeutic trial of carbamylglutamate also may help the diagnosis of NAGS deficiency [21, 30, 31]. Given the efficacy of carbamylglutamate in the treatment of NAGS deficiency, and the delays that can be involved with DNA testing, it has been suggested that a therapeutic trial be initiated for any patient with unexplained hyperammonemia [2, 25, 45, 46]. A therapeutic trial of carbamylglutamate would lead to faster recognition of NAGS deficiency, as well as improved outcomes due to the rapid reduction of ammonia levels associated with its use in NAGS deficiency cases.

While a response to carbamylglutamate aids in the diagnosis, it is not in itself diagnostic of NAGS deficiency, as carbamylglutamate has been shown to be effective in some cases of other inherited metabolic disorders by augmenting ureagenesis and decreasing plasma ammonia, including CPS1 deficiency, ornithine transcarbamylase (OTC) deficiency, citrullinemia type I (argininosuccinic acid synthetase deficiency), methylmalonic acidemia (MMA), propionic acidemia (PA), isovaleric acidemia (IVA), carbonic anhydrase VA (CAVA) deficiency, and multiple acyl-CoA dehydrogenase deficiency (MADD). Carbamylglutamate has been used in acute treatment of hyperammonemia in some cases of CPS1 deficiency [47–49], OTC deficiency [50], PA [51–55], MMA [51–56], IVA [57], CAVA deficiency [58, 59], and MADD [60] as well as long-term management of some cases of CPS1 deficiency [48, 61], citrullinemia type I [62], OTC deficiency [50], PA [63, 64], MMA [64], and MADD [60].

Proximal urea acid cycle disorders are currently included in the newborn screening programs of seven states, and the District of Columbia [65]. Given that the onset of NAGS deficiency can occur very early in life, before newborn screening results are available, and the morbidity and mortality from severe hyperammonemia in this time period is significant, there is concern that infants with early onset NAGS deficiency would not be identified early enough to fully benefit from newborn screening [2]. On the other hand, later onset cases are likely to benefit from early diagnosis and treatment. At the present time, newborn screening efforts are hampered by the instability of glutamine and low specificity and sensitivity of reduced citrulline levels [66, 67].

Outcomes were not presented in all published cases of NAGS deficiency. When described in the literature, outcome ranged from normal development to various levels of psychomotor or cognitive impairment, to death. Outcomes are likely related to the level and duration of hyperammonemia and the risk of further decompensation may be decreased by an appropriate dose of carbamylglutamate.

This literature review was limited by the amount of information presented in the case reports, case series, and other articles included. For example, we identified biochemical signs and clinical symptoms based on what was described in each article. Because not all authors provided information on all signs and symptoms, the data in Table 1 cannot be used to estimate the prevalence of the various signs and symptoms among NAGS deficiency cases.

Standardization of diagnosis and management of NAGS deficiency may improve care and outcomes for patients. Treatment with carbamylglutamate is the standard of care for NAGS deficiency. However, the literature indicates that dietary management is also part of care in some cases. Further studies of NAGS deficiency are needed to characterize management with carbamylglutamate and adjunct nutritional interventions. For example, the minimum dose of carbamylglutamate required to prevent hyperammonemia is not clear from the cases presented in the literature. More information about the triggers of hyperammonemic episodes among individuals treated with carbamylglutamate would be helpful in the identification of high-risk situations in which additional interventions (such as protein restriction or increased dose of carbamylglutamate) may be warranted. Finally, studies of the management of NAGS deficiency may help identify adjunctive nutritional interventions for times of illness or lack of access to carbamylglutamate.

## Conclusions

In conclusion, there are variations in the management of NAGS deficiency, such as maintenance dose of carbamylglutamate and the restriction of dietary protein. Prompt recognition and treatment of NAGS deficiency may improve outcomes of affected individuals. Our hope is that this literature review sheds light on the unmet needs of patients with NAGS deficiency, prompting novel research and optimization of care that improves outcomes for all patients.

## Supplementary information

**Additional file 1.** Summary of case reports of NAGS deficiency identified in the literature review.

## Data Availability

All data generated or analyzed during this study are included in this published article and its supplementary information files.

## Abbreviations

AAK: Amino acid kinase
ARG1: Arginase
ASL: Argininosuccinic acid lyase
ASS1: Argininosuccinic acid synthetase
AST: Aspartate aminotransferase
BUN: Blood urea nitrogen
CAVA: Carbonic anhydrase VA
CPS1: Carbamyl phosphate synthetase 1
GNAT: General control non-repressible 5 (GCN5)-related *N*-acetyltransferases
IVA: Isovaleric acidemia
MAAD: Multiple acyl-CoA dehydrogenase deficiency
MMA: Methylmalonc acidemia
NAG: *N*-Acetylglutamate
NAGS: *N*-Acetylglutamate synthase
ORNT1: Ornithine translocase
OTC: Ornithine transcarbamylase
PA: Propionic acidemia
