# Supplementary material for: Presentation and management of N-acetylglutamate synthase deficiency: a review of the literature
Source: Orphanet J Rare Dis. 2020 Oct 9;15:279. doi: 10.1186/s13023-020-01560-z (PMC7545900; doi:10.1186/s13023-020-01560-z)
Supplement: Supplementary file 1 — Additional file 1. Summary of case reports of NAGS deficiency identified in the literature review. [file 13023_2020_1560_MOESM1_ESM.docx]

**Supplemental Table 1: NAGS Deficiency Case Reports Literature Review**

Each row represents one patient.

| **Case No.**  **Age at presentation**  **(Relationship to other cases)** | **Presenting Symptoms** | **Plasma ammonia** | **Plasma Glutamine, Arginine, Alanine and Citrulline** | **Other abnormal test results** | **Liver NAGS enzyme activity** | **NAGS mutations and protein changes** | **Acute treatment** | **Long-term treatment** | **Outcome** | **References** |
| --- | --- | --- | --- | --- | --- | --- | --- | --- | --- | --- |
| **1.**  Neonatal | family history of neonatal death associated with hyperammonemia; hyperthermia, muscular hypertonus, respiratory insufficiency, abdominal distension; accidental withdrawal of benzoate led to coma, muscular hypotonia, and respiratory insufficiency | Presenting: 160-242 µmol/L (ref <80) | Glutamine: 1.02 mmol/L (ref 0.55 +/- 0.17)  Alanine: 0.36 mmol/L (ref 0.55 +/- 0.11)  Arginine: 0.12 mmol/L (ref 0.09 +/- 0.02) | Lysine: 0.28 mmol/L (ref 0.20 +/- 0.4)  Plasma Lactate: 3.6 (ref: <2.0)  Plasma urea nitrogen: low  Ornithine carbamyl transferase: 1.4 µmol/hour/g protein (ref: 9.5-33.3)  Hypocalcemia  coagulation problems | Not detectable (ref: 5.6-33 nmol/min/g protein)  (arginine stimulation not done) |  | IV benzoate (225 mg/kg), limited protein intake, nitrogen restriction (32 mM/kg/day), arginine (2.64 mM/kg/day) | carbamylglutamate (140-180 mg/kg/day);  arginine hydrochloride (0.7 g/kg/day) then switched to citrulline (430 mg/kg/day); protein 1.5 g/kg/day  hyperammonemia associated with increased protein intake or illness were treated with restriction of protein, high calorie nutrition, arginine and/or benzoate infusions | Gastric tube feeding up to 4.2 years of age; ataxia, hypotonia, and mental retardation at age 4.5 years. Deceased at age 9 years | Bachmann 1981; Bachman 1982; Zimmerman 1985; Schubiger 1991; Colombo 1994 (Patient 1) |
| **2.**  Neonatal | poor feeding, somnolence, tachypnea, vomiting, hypotonia, poor peripheral circulation | Presenting: 711 µmol/L | Glutamine: 640 µmol/L  Alanine: 340 µmol/L  Arginine: 30 µmol/L  Citrulline: Not detected | Respiratory alkalosis  Plasma methionine: low  Plasma ornithine: low | Not detectable  arginine stimulated = 0.44 nmol/min/g protein; (ref=22-52) |  | IV benzoate, arginine hydrochloride, glucose and insulin; and peritoneal dialysis (which was interrupted due to technical issues) | N/A | Deceased at age 8 days | Bachmann 1988 |
| **3.**  13 months; triggered by febrile illness | consanguinity, ataxia, tachycardia, shallow breathing, hypotonia, easily elicited deep tendon reflexes, pendulous patellar reflexes, facial palsy, enlarged liver, coma.  Weight and head circumference at the 3rd percentile | Presenting:  81 µmol/L (ref < 40)  Peak: 241 µmol/L (ref < 40) | Glutamine: 549 µmol/L (ref: 60-470)  Alanine: 477 µmol/L (ref: 100-310)  Citrulline: not detectable (ref: 10-30 µmol/L)  Arginine: 117 µmol/L (ref: 10-65) | Hyponatraemia (116 meq/1)  Hypokalaemia (2.5 meq/1)  natriuria (67 meq)  kaliuria (22 meq)  Plasma Proline: 220 µmol/L (ref: 50-190)  Plasma isoleucine: 23 µmol/L (ref: 25-95)  Plasma leucine: 44 µmol/L (ref: 45-155)  Plasma phenylalanine: 74 µmol/L (ref: 20-70)  Plasma lysine: 175 µmol/L (ref:45-145)  Urine alanine: elevated  Urine serine: elevated  Urine glycine: elevated  EEG: slow delta activity with triphasic waves,  CT scan: generalized cortical atrophy and slight ventricular dilatation. | Arginine stimulated = 47.9 nmol/min/g protein (ref: 144-320) |  | IV glucose, carnitine and vitamin B6; Oral feeding with a protein content of 0.5 g/kg per day was followed by recurrent vomiting and hyperammonaemia. IV sodium benzoate and arginine, oral neomycin and lactulose, were initiated. | N/A | Deceased 10 days after admission | Elpeleg 1990  (Patient IV-5) |
| **4.**  5 weeks  (older sibling of case 5) | family history of early childhood death, vomiting, history of constipation, diarrhea, lethargy, seizures, episodes of left upward lateral conjugate gaze with extension of arms and legs, tonic-clonic movements | Presenting: 215 µmol/L (ref: 18-47) | Glutamine: elevated  Arginine: low  Citrulline: low | Lactate: 6.1 mmol/L (ref: 0.6-2.0)  No arginino-succinic acid | undetectable |  | IV fluids and protein restriction (0.9 g/kg/day) | Ucephan (sodium benzoate/ sodium phenylacetate) at 250 mg/kg/day and protein restriction (1.5 g/kg/day) | normal growth at development at 18 months. Intermittent episodes of disconjugate gaze without associated seizure activity. | Pandya 1991  (Case 1) |
| **5.**  Neonatal  (younger sibling of case 4) | family history of hyperammonemia; vomiting, diarrhea, irritable, increased respiratory rate, malnourished, tachypnea, lethargy, poor response to stimuli, hypertonia. | Presenting: 185 µmol/L | Glutamine: elevated  Arginine: normal  Citrulline: normal | Bicarbonate low (8 mmol/L)  EEG suggestive of irritative encephalopathic process |  |  | IV fluids, sodium benzoate and arginine | Ucephan (sodium benzoate/ sodium phenylacetate) at 250 mg/kg/day and protein restriction (gradually increased from 05 to 0.9 g/kg/day) | at six months, development is at four months. Below 5^th^ percentile for weight. Head circumference at 10^th^ percentile. Mild appendicular spasticity. | Pandya 1991  (Case 2) |
| **6.**  2 months | history of vomiting and lethargy and hyperammonemia when switched from breast milk to cow’s milk at age two months; vomiting, lethargy, generalized involuntary movements, hypotonia, absent tendon reflexes, extensor plantar responses. Weight and head circumference at 3^rd^ percentile. | Presenting: 285 µmol/L | Alanine: 682 µmol/L  Glutamine: 755 µmol/L  Citrulline: 6 µmol/L | CT scan: generalized cortical atrophy;  brain MRI: increased signal intensity in the dentrum semiovale and initial cortical atrophy;  BAEP demonstrated an increased latency through the auditory pathway;  ERG was low | Arginine-stimulated = 55 nmol/min/g protein (ref:144-320) |  | sodium benzoate, arginine; protein restriction | sodium benzoate, arginine; protein restriction | profound mental retardation at 2 years and cortically blind; repeat MRI showed a marked cortical atrophy and increased hyperintensity of signal lesions in the deep white matter and centrum semiovale | Burlina 1992; Colombo 1994 (Patient 10) |
| **7.**  4 years, 10 months; triggered by minor upper respiratory illness | history of chronic diarrhea and poor appetite. Abdominal pain, vomiting, lethargy, decreased level of consciousness, mild cardiomyopathy | Presenting: 115 µg/dl (ref < 55) | Glutamine: 462.7 µmol/L (ref: 400-760)  Arginine: 111.2 µmol/L (ref: 23-117)  Citrulline: 29.0 µmol/L (ref: 10-20) | CPSI: 2.5  µmol/g liver/min (ref=2.9 +/-0.3)  OTC: 71  µmol/g liver/min (ref=80 +/- 6)  mild anemia | 110 nmol/g protein/min (control: 1160 +/- 750) |  | IV hydration and parenteral glucose administration and, subsequently, a protein restricted diet (< 1.5 g/kg/day), and neomycin and lactulose | sodium benzoate and sodium phenylacetate (250 mg/kg of each as a priming dose, then 250 mg/kg/day of each as a continuous infusion) with no oral feedings (could not tolerate); then when oral feedings could be tolerated, sodium phenylbutyrate (400 mg/kg/day divided into three doses), and then Ucephan (oral solution of 10% sodium benzoate and 10% sodium phenylacetate) to provide 250 mg/kg/day of each compound. |  | Vockley 1992 |
| **8.**  11 months | family history of NAGS deficiency; coma |  |  |  |  |  |  |  | Deceased at 13 months | Colombo 1994 (Patient 4) |
| **9.**  Neonatal | family history of NAGS deficiency |  |  |  |  |  |  |  | Growth retardation at 6 months | Colombo 1994 (Patient 6) |
| **10.**  Neonatal |  |  |  |  | 15% of normal level; not stimulated by arginine |  |  |  | Growth retardation at 1 month | Colombo 1994 (Patient 7) |
| **11.**  Neonatal |  |  |  |  | 10% of normal level; not stimulated by arginine |  |  |  | Normal growth at 4 months | Colombo 1994 (Patient 8) |
| **12.**  Neonatal | Coma |  |  |  | 34% of normal level; stimulated by arginine |  |  |  | Deceased at age 3 days | Colombo 1994 (Patient 9) |
| **13.**  27 years; triggered by pregnancy and caesarean delivery | history of episodic altered mental state and allergic reactions; coma shortly after awakening from anesthesia | Peak: 4781 μmol/l |  |  |  | c.935T>C  and  c.1292C>T  p.(L312P) and  p.(T431I) | sodium phenylbutyrate and sodium benzoate (discontinued due to patient preference); low-protein diet | oral contraceptives, then hysterectomy |  | Grody1994; Caldovic 2007 (No. 7); Sancho-Vaello 2016 (Family 9) |
| **14.**  Neonatal  (older sibling of case 42) | consanguinity, cyanosis, poor feeding, oliguria, trembling, truncal hypotonia, distal hypertonia, seizures, abnormal movements of lower limbs, lethargy, coma, and enlarged liver. | Presenting: 500 μmol/l | Glutamine 686 µmol/L (ref:822 +/- 274)  Citrulline 4 µmol/L (ref: 16 +/- 5)  Alanine: 445 µmol/L (ref: 255 +/- 78) | Plasma glutamic acid: 203 µmol/L (ref:90 +/- 65)  Plasma isoleucine: 6 µmol/L (low)  Cerebral ultrasound: cerebral edema.  CPS=0.66 µmol/h/mg protein (ref=1.84 +/- 0.5)  OTC=14.8 µmol/h/mg protein (ref=35.5 +/- 9.6)  EEG: poor with spikes | not calculable (<10%) (control: 34-203 nmol/min/g protein), not stimulated by arginine |  | peritoneal dialysis, IV sodium benzoate and arginine hydrochloride | carbamylglutamate 200 mg four times per day (at weight 10.68 kg). Then 80-100 mg/kg/day in three doses. | Normal growth at age one year | Guffon 1995; Guffon 2005  (Patient 1) |
| **15.**  5 months | central hypotonia, marked developmental delay, recurrent vomiting  NOTE: originally diagnosed as CPSI, but NAGS deficiency according to Sancho-Vaello 2016 | Peak: 260 µmol/L | Glutamine: 852-1025 µmol/L  Citrulline: slightly decreased  Arginine: slightly decreased | Urine orotic acid: 4.3 µmol/mmol creatinine (normal)  liver CPSI 0.29 µmol/hour/mg protein (15% of normal) | 79.8 nmol/min/g protein | c.598T>C  p.(C200R) | sodium benzoate and arginine, 1.5 g/kg/day protein; then carbamylglutamate | carbamylglutamate (4x200 mg), 3.5 g/kg/day protein | after one year of therapy, no further decompensation | Kuchler 1996; Sancho-Vaello 2016  (Family 4) |
| **16.**  20 years; triggered by febrile illness | consanguinity, history of hyperammonemia accompanying a febrile illness. Confusion, combative behavior, pneumonia, convulsions | Presenting: 78 µmol/L (ref <40)  Peak: 525 µmol/L | Glutamine: 1571 µmol/L (ref <718)  Citrulline: 2.2 µmol/L (ref >22) |  | 14.3 nmol/min per g protein (ref: 34-203)  Arginine stimulated = 21.4 nmol/min per g protein (ref: 144-320) |  | diazepam, sodium benzoate infusion (500 mg/kg per day), and 10% dextrose; Arginine (50 mg/kg per day) was given via a nasogastric tube; hemodialysis with high-calorie low-protein nasogastric feeding | carbamylglutamate 750 mg four times daily (60  mg/kg per day), and arginine (2.5 g/day; 50 mg/kg per day) | gross cerebral dysfunction and paraplegia and is incontinent of bladder and bowel. | Hinnie 1997 |
| **17.**  Neonatal | consanguinity, poor feeding, vomiting, cycling movements and fist clenching, hyperventilation | Presenting: 316 µmol/L | Glutamine: 1186 µmol/L  Citrulline: undetectable |  | 2.1 nmol/min/g protein  arginine stimulated = 2.5 nmol/min/g protein (62.8 in age-matched control) |  | IV glucose, sodium benzoate and L-carnitine. Then protein restriction (1 g/kg per day) and oral sodium benzoate (300 mg/kg per day). | oral carbamylglutamate (100 mg/kg per day in 4 divided doses); protein intake restriction (2.5 g/kg per day) and sodium benzoate (70 mg/kg per day). | at age 20 months, no neurological or developmental abnormalities | Morris 1998 |
| **18.**  12 years; triggered by high protein meal | history of one episode of vomiting, muscular hypotonia, somnolence and mild ketosis; aversion to protein-rich food. Vomiting, restless, disorientated and aggressive; ataxia; dilated pupils with a delayed response to light, atactic gait, exaggerated deep tendon reflexes, pyramidal signs were absent. | Presenting: 221 µmol/L (ref <53) | Glutamine: 835 µmol/L (ref: 254-823)  Cirulline: 12 µmol/L (ref: 1-46)  Arginine: 129 µmol/L (ref: 10-140) | Phenylalanine 98 umol/L (ref. 26-91)  LDH was mildly elevated (422 U/l)  EEG: diffuse slowing | 22 mU/mg protein (ref: >34).  arginine stimulated= 22 mU/mg protein (ref >144) | c.835G>A homozygous  p.(A279T) | protein withdrawal, a high energy intake (EQ 60) provided by a central venous line, IV L-arginine (2 mmol/kg within 2 h and 2 mmol/kg per day thereafter) and sodium benzoate (250 mg/kg as bolus and 250 mg/kg per day thereafter); restricted protein (0.7 g/kg per day, divided into 0.5 g/kg of natural protein and 0.2 g/kg of an essential amino acid mixture); Due to an oozing hepatic haemorrhage, she was given a 260 ml fresh blood transfusion, sodium benzoate was replaced by phenylbutyrate (340 mg/kg per-day) | carbamylglutamate (100 mg/kg/day in 4 doses), protein restricted to 1.2 g/kg/day | Normal cognitive function, and neurological findings at 24 years | Plecko 1998;  Haberle 2003a (Family 2);  Caldovic 2007 (No. 5); Sancho-Vaello 2016 ( Family 7) |
| **19.**  4 years  Note: later diagnosed with co-morbid porto-systemic shunt | history of periods of paroxysmal crying, unexplained lethargy, and refusing dairy and meat. Vomiting, lethargy, intention-tremor of the hands, ataxic gait. Length and head circumference at 10^th^ percentile. | Presenting: 229 µmol/L | Glutamine: 920 µmol/L (elevated) | Hypoglycaemia (2.0 mmol/L)  moderately elevated ASAT (90 IU/L)  elevated ALAT (70 IU/L)  elevated bilirubin (76 µmol/L)  low albumin (21.2 gr/L)  low fibrinogen (0.5 gr/L)  prolonged APTT of 43 sec  low blood urea (0.8 mmol/L)  low thrombocyte count (12 x 10^9^/L)  Low total (20 µmol/L) and free (12.6 µmol/L) carnitine plasma levels  Liver CPS = 9.7  mU/mg protein (reference >12 mU/mg protein). | 9 µU/mg protein (ref>34). Stimulated by arginine but still below reference range. |  | enteral low protein feeding. Then, carbamylglutamate (100 mg/kg/day in 6 doses) and arginine (200 mg four times a day). fed via a gastrostomy-catheter with a low protein mixture enriched in essential amino acids (total protein content 0.9 g/kg/day). | low protein diet (1 g/kg/d) and lactulose (20–30 g/d) | mental development was 2 standard deviations below that of age-matched controls. | Forget 1999; Dammers 2002 |
| **20.**  Neonatal  (older sibling of case 21) | consanguinity, unresponsive, convulsions | Presenting: 770 µM (normal: 9-32)  Peak: 1300 µM | Glutamine: 3654 µM (ref: <669)  Alanine: 3070 µM (ref: <484)  Citrulline: undetectable |  | 141 pmol/min/ mg protein (controls >34)  Arginine stimulated= 74 pmol/min/ mg protein (controls: 144–320) | 1036insC homozygous  p.(H346Pfs*10) | hemodialysis was initiated with subsequent administration of phenylacetate, citrulline, carnitine, and high caloric diet. | phenylacetate and citrulline and a high caloric diet. At 36 months, started carbamylglutamate (150mg/kg/day) and citrulline | developmental delay at age 4 years (walked unsteadily, failed to communicate, and did not recognize his relatives) | Elpeleg 2002 (proband);  Sancho-Vaello 2016 (Family 37) |
| **21.**  Neonatal  (younger sibling of case 20) | consanguinity, family history of hyperammonemia, unresponsive, coma | Presenting: 287 µM (ref: 9-32)  Peak: 1900 µM | Glutamine: 1732 µM (ref: <669)  Citrulline: undetectable |  |  | 1036insC homozygous  p.(H346Pfs*10) | hemodialysis, IV phenylacetate, enteral citrulline | At age three months, started carbamylglutamate (150mg/kg/day) | At 2 years of  age, he had no words, muscle tone was markedly increased, and there were no voluntary movements. | Elpeleg 2002 (brother of proband);  Sancho-Vaello 2016 (Family 37) |
| **22.**  12 years; triggered by eating a hamburger | history of attention deficits and learning disabilities, episodes of anxiety and irritability, and hand tremor. Acute headache, vomiting, confusion, depressed consciousness. | Presenting: 350 µmol/l (ref<50 µmol/l) | Glutamine 801 µmol/l (normal <700 µmol/l)  Citrulline 21 µmol/l (ref 30–50 µmol/l)  Arginine 37 µmol/l (ref 60–120 µmol/l)  Alanine: normal | EEG: focal discharges from the temporal lobe.  Cerebral axial tomography: bitemporal patchy oedema.  MRI: hyperintense areas in both insulae. | 30 µIU/mg of protein (34 µIU in a control patient)  Arginine stimulated = 46 µIU/mg of protein (144 µIU in the control patient). |  | benzoate (0.25 g/kg per day) and phenylbutyrate (0.5 g/kg per day); carbamylglutamate loading test | carbamylglutamate (15 mg/kg per day) and a diet with 70 g protein/day |  | Bélanger-Quintana 2003 |
| **23.**  Neonatal | grunting, jitteriness, tachypnea, hypotonia, hyperthermia | Not available at hospital | Glutamate: elevated  Alanine: elevated | Urine citrulline: undetectable  Urine arginine-succinate: undetectable  Alkalosis | 10 U (normal >34  arginine stimulated = 20 U (normal >144) | c.971G>A  (presumed homozygous based on parents’ genotypes)  p.(W324*) | N/A (diagnosis was post-mortem) | N/A | Deceased at age 4 days | Caldovic 2003 (Family 1); Sancho-Vaello 2016 (Family 34) |
| **24.**  Neonatal | hyperammonemic encephalopathy | Peak: 942 μmol/l |  |  | 6 pmol/min/g protein (ref >34)  Arginine stimulated=14 pmol/min/g protein (ref>144) | c.1306_1307 insT and  IVS3-2A→T  p.T439Hfs*52  and  p.V306Gfs*26 |  |  | Death at day 6 | Haberle 2003a  (Family 1); Haberle 2003b; Sancho-Vaello 2016 (Family 32) |
| **25.**  Neonatal | consanguinity, hyperammonemic encephalopathy |  |  |  | 14 pmol/min/g protein (ref >34)  Arginine stimulated= 9 pmol/min/g protein (ref>144) | c.1289T>C  (presumed homozygous based on parents’ genotypes)  p.L430P |  |  | Death at day 4 | Haberle 2003a  (Family 3); Sancho-Vaello 2016 (Family 16) |
| **26.**  Neonatal  (older sibling of case 27) | consanguinity, hyperammonemia | Presenting: 2000 μmol/l |  | CPSI (20% of reference  limit) | 78 pmol/min/mg protein (ref >34).  arginine stimulated = 104 pmol/min/mg protein (ref >144) | c.971G>A homozygous  p.(W324*) |  |  | Deceased age 22 days | Haberle 2003a  (Family 4); Gessler 2010  (Case 2 (TD));  Sancho-Vaello 2016 (Family 33) |
| **27.**  Neonatal  (younger sibling of case 26) | consanguinity, family history of neonatal death with hyperammonemia; repeated hospitalizations due to vomiting and increased ammonia | Presenting: 138-149 μmol/l (ref <100)  Peak: 393 μmol/l | Glutamine: 1,332 and 1,961μmol/l (ref:254–823) | Leucine: 33μmol/l (ref: 56–98)  Isoleucine: 7μmol/l (ref: 31–48) | 98 pmol/min/mg protein (ref >34).  arginine stimulated = 64 pmol/min/mg protein (ref >144) | c.971G>A homozygous  p.(W324*) | parenteral high caloric diet with arginine, then switched to breast milk with sodium benzoate and arginine supplementation administered orally. | carbamylglutamate (200 mg/kg per day) and arginine (250 mg/kg per day). Gradually, carbamylglutamate reduced to 10 mg/kg/day, which was associated with increased ammonia. Then increased to 15 mg/kg/day. | Normal development at age 13 years | Haberle 2003a  (Family 4); Gessler 2010  (Case 2 (YD))  Sancho-Vaello 2016 (Family 33) |
| **28.**  Neonatal  (sibling of case 29, relative of case 30) | consanguinity, coma |  |  |  |  | c.1450T>C  homozygous  p.(W484R) |  |  | Deceased at month 6 | Haberle 2003a  (Family 5); Sancho-Vaello 2016 (Family 20) |
| **29.**  (sibling of case 28, relative of case 30) | consanguinity, family history |  |  |  |  | c.1450T>C  homozygous  p.(W484R) |  | carbamylglutamate |  | Haberle 2003a  (Family 5); Sancho-Vaello 2016 (Family 20) |
| **30.**  (relative of cases 28 and 29) | consanguinity, family history |  |  |  |  | c.1450T>C  homozygous  p.(W484R) |  | carbamylglutamate |  | Haberle 2003a  (Family 5); Sancho-Vaello 2016 (Family 20) |
| **31.**  Neonatal | poor feeding, lethargy |  |  |  | 0 pmol/min/g protein (ref >34)  Arginine stimulated= 72 pmol/min/g protein (ref>144) | c.1299G>C  (presumed homozygous based on parents’ genotypes)  p.(E433D) |  |  | Deceased at month 3 | Haberle 2003a  (Family 6); Sancho-Vaello 2016 (Family 18) |
| **32.**  Neonatal  (older sibling of case 33) | lethargy, anorexia, and respiratory distress, progressing to coma and generalized tonic-clonic seizures. | Peak: 1700 µmol/L (ref: 64-107) | Glutamine: 1268 µmol/L (ref: 538–958)  Citrulline: 0 µmol/L (ref: 8–29)  Arginine: 26 µmol/L (ref: 22–88) | Serum glutamine: 362 µmol/L (ref: 0-50)  Serum ornithine: 47 µmol/L (ref: 49–151)  serum argino-succinate: undetectable  Liver CPSI activity was 1.4 U (control 3.8 U), OTC activity was 35 U (control 94 U)  MRI: bilateral low signal intensity in the globi pallidi and high signal intensity in the corpora striata and white matter subjacent to the insular and perirolandic cortices. Intraventricular hemorrhage and retrocerebellar subdural hematoma |  | c.1025delG homozygous  p.(R342Pfs*50) | hemodialysis; IV sodium benzoate, sodium phenylacetate, and arginine hydrochloride | cadaveric liver transplantation | truncal hypotonia, appendicular hypertonia, choreoathetosis, and global neuro-developmental delays | Takanashi 2003  (Case 1); Caldovic 2003 (Family 2 Patient 1); Sancho-Vaello 2016 (Family 36) |
| **33.**  Neonatal  (younger sibling of case 32) | family history of hyperammonemia**;** lethargy, anorexia, vomiting, respiratory distress, clonic arm jerking, seizures. | Peak: 978 µmol/L (ref: 64-107) | Glutamine: 1710 µmol/L (ref: 538-958)  Citrulline: 0 µmol/L (ref: 8-29)  Arginine: 24 µmol/L (ref: 22-88) | Serum argino-succinate: undetectable  Serum ornithine: 48 µmol/L (ref: 49-151)  Hepatic CPSI enzyme activity was 1.3 U (control 5.9 U), and OTC activity was 105 U (control 147 U).  MRI: T1 shortening in the bilateral lentiform nuclei (the globi pallidi more than the putamina) and the deep sulci of insular and perirolandic regions, T2: high signal intensity in the medial putamina, and white matter subjacent to insular and perirolandic cortices, as well as heterogeneous high and low signal intensity in the globi pallidi. |  | c.1025delG homozygous  p.(R342Pfs*50) | required intubation, anticonvulsant therapy, 34 hours of hemodialysis and medical therapy | cadaveric liver transplantation | non-oral food intake at 6 weeks; otherwise, normal development at 6 weeks | Takanashi 2003  (Case 2); Caldovic 2003 (Family 2 Patient 2);  Sancho-Vaello 2016 (Family 36) |
| **34.**  Neonatal  (older sibling of case 52) | consanguinity, seizures, coma | Presenting: 926 µmol/L  Peak: 1187 µmol/L |  | MRI at 1 week of age showed edema, most pronounced in the border between white and grey matter |  | c.1241G>C  homozygous  p.(R414P) | IV glucose infusion as well as sodium benzoate and arginine. No oral feeds were given. Treatment with carbamylglutamate 200 mg/kg per day in 3 divided doses was given orally | carbamylglutamate 50 mg/kg/day. Until 5 months of age the boy was breast fed without protein restriction. With the introduction of foods, 2 g/kg per day protein restriction was introduced. | normal development at 2.5 years of age | Alm 2004; Nordenstrom 2007; Sancho-Vaello 2016 (Family 15) |
| **35.**  Neonatal  (older sibling of case 36) | vomiting, irritability, lethargy, dehydration | 256 µmol/l (ref<35) | Glutamine: markedly elevated  Alanine: markedly elevated  Citrulline: normal | Metabolic acidosis then after infusion of fluids, developed a respiratory alkalosis  CPSI  2.3 µmol/mg/gm (control 4.1) and OTC 31.5 (control 78.6) |  | c.1097–1G>C and c.1526G>A  IVS4-1 and p.(R509Q) |  | protein restricted diet, sodium benzoate, and L-citrulline | hyperactivity, headaches, irritability, and hallucinations. | Caldovic 2005  (Case 1);  Sancho-Vaello 2016 (Family 26) |
| **36.**  9 years  (younger sibling of case 35) | lethargy, anorexia, vomiting, respiratory distress, and seizures. | Peak: 978 µmol/l | Glutamine: 1,710 µmo/l (ref: 254–823)  Citrulline: undetectable (ref: 1–46) | CPSI enzyme activity  was 1.3 units (control 5.9) and OTC activity was 105 units  (control 147) |  | c.1097–1G>C and c.1526G>A  IVS4-1 and p.(R509Q) | hemodialysis |  |  | Caldovic 2005  (Case 2);  Sancho-Vaello 2016 (Family 26) |
| **37.**  33 years; triggered by vehicle accident | combativeness, confusion, and tonic clonic seizures. | Presenting: 400 µmol/l  Peak: 621 µmol/l |  | AST: 87 U/l (ref: 4–40)  creatinine kinase: 1857 U/l (ref: 30–210)  Electro-encephalogram: diffuse slowing |  | c.518T>A and c.1292C>T  p.(V173E) and p.(T431I) | dialysis, phenylbutyrate (via nasogastric tube) and IV Na-benzoate and L-arginine-HCl. | N/A | Deceased | Caldovic 2005  (Case 3); Sanco-Vaello 2016 (Family 2) |
| **38.**  Neonatal | irritable with poor feeding, hypotonia, drowsiness, tremor, and no suck | Presenting: 182 µmol/L (controls <50) |  |  | decreased |  | carbamylglutamate |  | developing normally | Guffon 2005  (Patient 2) |
| **39.**  3 months  (sibling of case 40) | vomiting and progressive altered conscious state, leading to seizures and coma. | Presenting: 2368 µmol/L (controls <50) |  |  | decreased |  | sodium benzoate, hemodiafiltration; then carbamylglutamate | N/A | deceased | Guffon 2005 (Patient 3) |
| **40.**  Neonatal  (sibling of case 41) | family history of neonatal death with hyperammonemia; hyperammonemia | Presenting: 127 µmol/L (controls <50)  Peak: 133 µmol/L |  |  | decreased |  | one dose of carbamyl-glutamate (166 mg/kg); one hour later, a second dose of carbamylglutamate (83 mg/kg) was administered. |  | developing normally | Guffon 2005  (Patient 4) |
| **41.**  3 months; triggered by introduction of cow’s milk | vomiting and weight loss, severe axial hypotonia, hyporeactivity, and hepatomegaly. | Presenting: 96 µmol/L (controls <50)  Peak: 367 µmol/L |  |  | decreased |  | carbamylglutamate |  | developing normally | Guffon 2005  (Patient 5) |
| **42.**  Neonatal  (younger sibling of case 14) | consanguinity, family history of carbamylglutamate-responsive hyperammonemia |  |  |  | decreased |  | carbamylglutamate |  | developing normally | Guffon 2005 (Patient 6) |
| **43.**  3 months  (younger sibling of case 44) | hypotonia, hepatomegaly, failure to thrive, hyperammonemia |  |  |  |  | c.598T>C  homozygous  p.(C200R) |  |  |  | Schmidt 2005  (Family 1);  Sancho-Vaello 2016 (Family 3) |
| **44.**  (Older sibling of case 43) | asymptomatic |  |  |  |  | c.598T>C  homozygous  p.(C200R) |  |  |  | Schmidt 2005  (Family 1);  Sancho-Vaello 2016 (Family 3) |
| **45.**  Neonatal  (older sibling of case 46) | poor feeding, seizures, lethargy |  |  |  |  | c.1228T>C  homozygous  p.(S410P) |  |  | Normal at one years | Schmidt 2005  (Family 2);  Sancho-Vaello 2016 (Family 13) |
| **46.**  (younger sibling of case 45) | family history |  |  |  |  | c.1228T>C  homozygous  p.(S410P) |  | carbamylglutamate |  | Schmidt 2005  (Family 2);  Sancho-Vaello 2016 (Family 13) |
| **47.**  Neonatal |  |  |  |  |  | c.1289T>C  homozygous  p.(L430P) |  |  |  | Schmidt 2005  (Family 3) |
| **48.**  Neonatal |  |  |  |  |  | c.1450T>C  homozygous  p.(W484R) |  |  |  | Schmidt 2005  (Family 4) |
| **49.**  Neonatal | hypotonia, poor feeding, lethargy |  |  |  |  | c.1552G>A  homozygous  p.(A518T) |  |  |  | Schmidt 2005  (Family 5); Sancho-Vaello 2016 (Family 28) |
| **50.**  40 years | frequent migraine headaches, intermittent staring spells,  nausea, recurrent vomiting, lethargy, and ataxia followed by coma | Peak: 500 µM | Glutamine: increased |  | 5%  (arginine stimulation: 4%) | c.1048G>A and c.1324C>G  p.(V350I) and p.(L442V) | protein diet and lactulose | low protein diet, lactulose, Na phenylbutyrate (Buphenyl) and L-citrulline as well as ondansetron for chronic nausea. Then carbamylglutamate | high carbohydrate and low protein diet resulted in obesity and type II diabetes requiring insulin | Caldovic 2007  (No. 11); Tuchman 2008; Sancho-Vaello 2016 (Family 10) |
| **51.**  Late-onset | seizures and coma |  |  |  | 50%  (arginine stimulation: 75%) | c.1048G>A and c.1324C>G  p.(V350I) and p.(L442V) |  |  |  | Caldovic 2007  (No. 18) |
| **52.**  (younger sibling of case 34) | consanguinity, family history |  |  |  |  | c.1241G>C  homozygous  p.(R414P) |  | carbamylglutamate |  | Nordenstrom 2007; Sancho-Vaello 2016 (Family 15) |
| **53.**  Neonatal | vomiting, feeding intolerance and episodic confusion | Peak: 500 μM | Arginine: normal  Citrulline: normal |  | decreased | c.278delC and c.499A>G |  | initially treated with carbamylglutamate (140 to 23 mg/kg/d). Then, the dose of 200 mg/d was kept unchanged for about 4 years. At age 20 years, carbamylglutamate is 800 mg/d, with a normal diet. | Normal development at age 20 years | Corne 2011 |
| **54.**  10 years | nausea, ataxia, somnolence | Presenting: 203 µM | Glutamine: 868 µM  Citrulline: 12 µM | BUN 3.6 mM/L |  | -3063C>A homozygous |  | protein restriction **(**0.5g/kg/d) and sodium phenylbutyrate (18g/day). Then after success with a three-day clinical trial of carbamyl-glutamate, it was continued (2.2 g/m^2^/d) |  | Heibel 2011 |
| **55.**  38 years | 20-year history of fluctuating behavioral changes associated with nausea and vomiting. Vomiting, headache, confusion and bizarre behavioral changes, mild spasticity on assessment of muscle tone with brisk reflexes, sustained ankle clonus, and down-going plantar reflexes. Disinhibition and fluctuating drowsiness. Impaired coordination, asterixis. | Presenting: 434 µmol/L (ref 15–55) | Glutamine: 1062 μmol/L (ref: 109–750)  Citrulline: 15 μmol/L (ref: 10–50) | respiratory alkalosis  EEG: severe generalized encephalopathy with associated triphasic waves |  | E433G and IVS6+5G>A | IV fluids, lactulose and a relatively lower protein diet. | low protein diet (around 1 gm/kg), sodium phenylbutyrate 200 mg/kg TID and citrulline 50mg/kg TID; then after diagnosis, carglumic acid 1200 mg TID (about 150 mg/kg/day) | short term memory loss | Cartegena 2013 |
| **56.**  Neonatal | hyperammonemia | Presenting: 328 mmol/L (typo?) |  |  |  | c.1450T>C (presumed homozygous)  p.(W484R) | Discontinuation of enteral feeding, IV glucose, oral sodium benzoate (200 mg/kg/day) and arginine (200 mg/kg/day). Then carbamylglutamate was started with a dose of 100 mg/kg/day | carbamylglutamate (10 – 50 mg/kg/day fluctuations with growth and dose adjustments – final = 30 mg/kg/day) | At 3 years of age, the patient has no neuro-developmental abnormalities. | Kiykim 2014 |
| **57.**  Neonatal | consanguinity, lethargy, poor feeding and vomiting, supraventricular tachycardia, mild hypotonia with incomplete Moro, poor sucking reflex | Presenting: 290 µmol/l (ref: <100) | Glutamine: 1004 μmol/L (elevated)  Citrulline: normal  Arginine: normal | Respiratory alkalosis |  | c.1450T>C homozygous  p.(W484R) | eliminate protein, infusion of 20% glucose (110 kcal/kg/day), sodium benzoate (loading dose of 350 mg/kg, followed by 350 mg/kg/d) and arginine hydrochloride (loading dose of 250 mg/kg, followed by 250 mg/kg/d). Then carbamylglutamate was added (100 mg/kg/d). | carbamylglutamate (100 mg/kg/d in four divided doses) | Normal growth and development at nine months | Van Leynseele 2014; Sancho-Vaello 2016 (Family 25) |
| **58.**  Neonatal | poor feeding, vomiting and lethargy | Presenting: 800 µmol/L (ref: 11-32) | Glutamine: 1117 μmol/L (ref: 376–709)  Arginine: 51 μmol/L (ref: 6–140)  Citrulline: 7 μmol/L (ref: 10–45) |  |  | c.929T>C and c.1464_1465  del  p.(V310A) and p.(H488Qfs*2) | IV fluids, protein restriction and peritoneal dialysis | low-protein diet (1.0 g kg−1 per day) with essential amino acids, sodium benzoate (250mg/kg per day) and carnitine (100 mg/kg per day) and citrulline (100mg/kg per day). Then, at age 8 years, carbamylglutamate (100 mg/kg in four divided doses, then reduced to 75mg/kg per day) | poor growth, seizures, mild mental retardation and attention-deficit/ hyperactivity disorder at age 9 years. | Kim 2015 |
| **59.**  Neonatal | poor sucking, decreased activity, lethargy, tachypnea, and convulsions | Presenting: 387 μM  Peak: 1194 μM | Glutamine: elevated  Citrulline: undetectable | Glycine: elevated  respiratory alkalosis |  | c.1097-2A>T homozygous | Ammonul® (sodium phenylacetate and sodium benzoate), IV arginine, IV high dextrose, and continuous insulin infusion; peritoneal dialysis. Carbamylglutamate was subsequently initiated after about 24 hours, with discontinuation of dialysis and insulin infusion. | oral citrulline (300 mg/kg/day) and sodium benzoate (300 mg/kg/day) and protein restricted (2 g/kg/day) formula composed of a mixture of essential amino acid formula and regular infant formula. Carbamyl-glutamate intake was discontinued due to limited availability and rarity of NAGS deficiency. Then after diagnosis (3 weeks after discharge), carbamylglutamate (200 mg/kg/day) and regular infant formula |  | Al Kaabi 2016 |
| **60.**  Neonatal | vomiting, poor feeding, episodic confusion |  |  |  |  | c.499A>G  c.278delC  p.(M167V) and p(P93Qfs*18) |  |  | Normal at 20 years | Sancho-Vaello 2016  (Family 1) |
| **61.**  Neonatal | hyperammonemia |  |  |  |  | c.779C>T  homozygous  p.(P260L) |  |  |  | Sancho-Vaello 2016  (Family 5) |
| **62.**  Neonatal | vomiting, failure to thrive,  hepatomegaly, recurrent asymptomatic  hyperammonemia |  |  |  |  | c.791C>T  homozygous  p.T264M |  | benzoate,  citrulline, protein restriction | Normal at 5 years | Sancho-Vaello 2016  (Family 6) |
| **63.**  Late-onset  (relative of cases 64, 65 and 66) | “mild course” |  |  |  |  | c.872T>A  homozygous  p.(I291N) |  |  | Normal without carbamyl-glutamate | Sancho-Vaello 2016  (Family 8, member 1) |
| **64.**  Late-onset  (relative of cases 63, 65 and 66) | “mild course” |  |  |  |  | c.872T>A  homozygous  p.(I291N) |  |  | Normal without carbamyl-glutamate | Sancho-Vaello 2016  (Family 8, member 2) |
| **65.**  Late-onset  (relative of cases 63, 64 and 66) | “mild course” |  |  |  |  | c.872T>A  homozygous  p.(I291N) |  |  | Normal without carbamyl-glutamate | Sancho-Vaello 2016  (Family 8, member 3) |
| **66.**  Late-onset  (relative of cases 63, 64 and 65) | “mild course” |  |  |  |  | c.872T>A  homozygous  p.(I291N) |  |  | Normal without carbamyl-glutamate | Sancho-Vaello 2016  (Family 8, member 4) |
| **67.**  2 months | family history of early childhood death; severe hyperammonemia |  |  |  |  | c.1172T>G  c.1450T>C  p.(L391R) and p.(W484R) |  | carbamylglutamate | Death after stopping carbamyl-glutamate | Sancho-Vaello 2016  (Family 11) |
| **68.**  15 years | somnolence, decreased consciousness | Peak: 350 µmol/L |  |  |  | c.1192A>T  2^nd^ allele not found  p.(S398C) |  |  | Normal | Sancho-Vaello 2016 (Family 12) |
| **69.**  Neonatal | vomiting, lethargy |  |  |  |  | c.1228T>C  homozygous  p.(S410P) |  |  | Deceased at day 20 | Sancho-Vaello 2016  (Family 14) |
| **70.**  Neonatal |  |  |  |  |  | c.1289T>C  homozygous  p.L430P |  |  |  | Sancho-Vaello 2016  (Family 17) |
| **71.**  Neonatal |  |  |  |  |  | c.1370G>A  homozygous  p.G457D |  |  | Deceased at day 5 | Sancho-Vaello 2016  (Family 19); Haberle 2003a (Family 5) |
| **72.**  Neonatal | coma |  |  |  |  | c.1450T>C  homozygous  p.(W484R) |  |  | Deceased at 6 months | Sancho-Vaello 2016  (Family 20) |
| **73.**  Neonatal | poor feeding, seizures |  |  |  |  | c.1450T>C  homozygous  p.(W484R) |  | carbamylglutamate | Psychomotor delay and spasticity | Sancho-Vaello 2016  (Family 21) |
| **74.**  Neonatal | hyperammonemia |  |  |  |  | c.1450T>C  homozygous  p.(W484R) |  |  |  | Sancho-Vaello 2016  (Family 22) |
| **75.**  Neonatal |  |  |  |  |  | c.1450T>C  homozygous  p.(W484R) |  | “Standard treatment for hyperammonemia without NCG” |  | Sancho-Vaello 2016  (Family 23) |
| **76.**  Neonatal | hyperammonemia |  |  |  |  | c.1450T>C  homozygous  p.(W484R) |  |  |  | Sancho-Vaello 2016  (Family 24) |
| **77.**  16 years | Reye-like syndrome with hyperammonemia |  |  |  |  | c.1535A>G  2^nd^ allele not found  p.Y512C |  |  | Normal | Sancho-Vaello 2016  (Family 27) |
| **78.**  4 years | convulsions and microcephaly |  |  |  |  | c.1552G>A  p.(A518T) |  |  | microcephaly | Sancho-Vaello 2016  (Family 29) |
| **79.**  Neonatal | coma |  |  |  |  | c.545delC  p.(A182Vfs*23) |  |  | Deceased at 72 hours | Sancho-Vaello 2016  (Family 31) |
| **80.**  Neonatal  (sibling of case 81) | coma |  |  |  |  | c.991C>T  homozygous  p.(Q331*) |  |  | Deceased on day 7 | Sancho-Vaello 2016  (Family 35 index patient) |
| **81.**  Neonatal  (sibling of case 80) | coma |  |  |  |  | c.991C>T  homozygous  p.(Q331*) |  |  | Deceased on day 19 | Sancho-Vaello 2016  (Family 35 sibling) |
| **82.**  No data given |  |  |  |  |  | c.1264G>T  homozygous  p.(E422*) |  |  |  | Sancho-Vaello 2016  (Family 38) |
| **83.**  Neonatal | hyperammonemia |  |  |  |  | c.1313dup  homozygous  p.(T439Hfs*52) | dialysis | carbamylglutamate |  | Sancho-Vaello 2016  (Family 39) |
| **84.**  Neonatal | vomiting and feeding problems; family history of neonatal death |  |  |  |  | c.1313delG  homozygous  p.G438Afs*10 |  | carbamylglutamate | Normal at 1 year | Sancho-Vaello 2016  (Family 40) |
| **85.**  Neonatal | hyperammonemia | Peak: 368 μmol/L |  |  |  | c.1494G>A  2^nd^ allele not found  p.W498* |  | carbamylglutamate |  | Sancho-Vaello 2016  (Family 41) |
| **86.**  Neonatal | family history of death due to hyperammonemia; poor feeding, sleepiness, vomiting, lethargy, and respiratory distress; liver was palpable (1.5 cm below the costal margin) with truncal hypotonia and spasticity in extremities. seizures occurred during hospitalization. | Presenting: 920 μmol/L (ref. < 100)  Peak: 3556 μmol/L | Glutamine: 1628.6 μmol/L (ref: 410-960)  Citrulline: 6.6 μmol/L (ref: 8-47)  Arginine: 26 μmol/L (ref: 20-160)  Alanine: 1151.5 μmol/L (ref: 200-600) | Lactate: 80 mg/dl (ref:0.9-20)  Glutamate 312.9 μmol/L (ref: 10-190)  metabolic acidosis  Brain ultrasound showed brain edema. |  | c.1172T>G and c.1450T>C  p.(L391R) and p.(W484R) | oral feeding was stopped and 12.5% glucose infusion (120 kcal/kg per day) was started; IV L-arginine hydrochloride (600 mg/kg/d) and sodium benzoate as well as oral sodium phenyl butyrate (both with loading doses 500 mg/kg followed 500 mg/kg/d) by nasogastric tube was started; peritoneal dialysis | high doses of oral sodium phenylbutyrate, sodium benzoate, and L-arginine with UCD formula. Then at 3 months, carbamylglutamate (100 mg/kg/d in 3 doses) with protein intake of 2-2.5 g/kg/day. Parents discontinued carbamylglutamate at 4.5 months; then treated for five days (until death) with high dose sodium benzoate, sodium phenylbutyrate, L-arginine hydrochloride and protein restriction. | At 3 months, failure to thrive with weight (<5%), head circumference (<5%), and length (<5%); truncal hypotonia with spasticity in extremities, could not control his head and did not have normal gaze. Brain CT scan and MRI showed marked brain atrophy, bilateral low attenuated white matter of centrum of semi-oval and peri-ventricular areas. Deceased at 4.5 months | Sayarifard (2016) |
| **87.**  Neonatal | respiratory distress, hypertonia, unresponsive to pain, respiratory failure, coma | Presenting: 2,235 µmol/l (ref: <100)  Peak: 2455 µmol/l | 30 min after initial treatment:  Glutamate: 872 µmol/l (Ref: 400–850)  Citrulline: undetectable  Arginine: 938 µmol/l, (ref: 17–120) | CRP peak: 20 mg/L (ref: <5)  blood glucose of 1.1 mmol/l (normal >=2.2 mmol/l) |  | c.971G>A homozygous  p.(W324*) | sodium benzoate, sodium phenylacetate, L-arginine, and carbamylglutamate | carbamylglutamate (40 mg/kg/day) | Normal development at 7 months | Reigstad 2017 |
| **88.**  59 years; triggered by fractured pelvis | confusion, isocoric pupils, and pareses of both  arms and legs, coma | Presenting: 280 µM | Glutamine: 777 µM  Alanine: 449 µM  Citrulline: 33 µM  Arginine: 52 µM | respiratory alkalosis |  | c.603G>C (presumed homozygous)  p.(K201N) | IV high dose glucose, laxatives, and sodium benzoate. | protein restriction (0.5 g per kg bodyweight per day) and sodium benzoate. Then carbamylglutamate (600 mg bid) and protein restriction (0.8 g per kg per day) |  | Van de Logt 2017 |
| **89.**  52 years; triggered by period of insomnia, low caloric intake and dehydration | consanguinity, history of headaches, history of post-partum psychosis, self-selected low-protein diet, confusion, psychomotor agitation, nausea, and vomiting. Performed repetitive and senseless actions | Presenting: 45 µmol/L (ref: 11-32)  Peak: 330 µmol/L |  | Video EEG monitoring showed bilateral frontal epileptiform and slow activity. Brain MRI showed hyper-intensities in the cortical frontal regions. |  | c.344C>T homozygous  p.A115V | protein intake was stopped and glucose IV infusion and oral supplementation with L-arginine (195 mg/kg/day) were started. Then, sodium benzoate 160 mg/kg/day in 4 divided doses was introduced. | carbamylglutamate at 16 mg/kg/day in 4 doses was started, then increased to 24 mg/kg/day. | none | Cavicchi 2018 |
| **90.**  29 years; triggered by childbirth | delirium and ataxia | Presenting: > 200 µmol/L | Glutamine: elevated  Alanine: elevated  Citrulline: normal | Lysine: elevated  during dialysis, developed hemolytic anemia and mild elevation of liver function tests |  | c.1080G>C and  c.426+3 G>C  p.(E360D) | hemodialysis, nitrogen scavengers, citrulline supplementation and a protein-restricted diet | carbamylglutamate |  | McNutt 2018 |
| **91.**  3 years, 9 month | avoidance of milk and dairy products, fish and meat; encephalopathy; restless, drowsy/lethargic, and repeating incomprehensible sentences followed by increasing loss of consciousness | Presenting: 314 μmol/L (ref <50)  Peak: 462 μmol/L | Glutamine: 482 μmol/L (ref: 400–720)  Alanine: 1043 μmol/L (ref: 176–480)    Citrulline: 10 μmol/L (ref: 17–50)  Arginine: 17 μmol/L (ref: 32–128) | Isoleucine: decreased  Leucine: decreased  Valine: decreased  EEG was suspicious for focal epilepsy | 94 μIE/mg protein (ref. >34)  Arginine-stimulated = 197 μIE/mg protein (ref. > 144) | homozygous c.-3026C>T due to partial maternal uniparental disomy of chromosome 17 | High caloric drip, without protein. IV arginine (250 mg/kg/d), sodium benzoate and sodium phenylacetate (250 mg/kg/d). After 3 days, nitrogen scavengers were increased to 400 mg/kg/d and carbamylglutamate started simultaneously (5 × 200 mg/d). Reintroduction of protein at final dose of 1 g/kg/d. | carbamylglutamate |  | Williams 2018 |
| **92.**  6 years;  febrile illness  (dizygotic twin of case 93) | vomiting with slow speech, anxiety, confusion, disorientation and altered consciousness; history of poor feeding and problematic eating habits with rejection of meat and fish. Weight and height were at the 3rd percentile. | Presenting: 251 μmol/L (ref: 0–35) | Glutamine: 895 μmol/L (ref: 400–720)  Alanine: 719 μmol/L (ref: 176–480) |  |  | c.-3026C>T  homozygous | IV glucose fluids, sodium benzoate. After a second episode 3 months later, carbamylglutamate was initiated at 100 mg/kg/d. | carbamylglutamate (50 mg/kg/d) and protein restriction (1.8 g/kg/d) | Weight and height reached the 50th percentile and the neurological and developmental assessments were normal. | Williams 2018 |
| **93.**  6 years  (dizygotic twin of case 92) | family history of NAGS deficiency; history of poor appetite and avoidance of foods with high protein content; weight and height were at 5th percentile. | Presenting: 85 μmol/L (ref < 35) | Glutamine: 1109 μmol/L (ref: 400–720) |  |  | c.-3026C>T  homozygous |  | carbamylglutamate (50 mg/kg/d) |  | Williams 2018 |
| **94.**  Neonatal | consanguinity, family history of death due to hyperammonemic encephalopathy |  |  |  |  | c.991C>T homozygous  p.(Q331*) |  |  | Deceased | Bijarnia-Mahay 2018 |
| **95.**  Neonatal | family history of sibling death; hyperammonemic encephalopathy |  |  |  |  | c.787G>T homozygous  p.(E263*) |  |  | Deceased | Bijarnia-Mahay 2018 |
| **96.**  Neonatal  (older sibling of case 97; cousin of case 98) | hypotonia, weight loss, alkalosis, hyperlactatemia | Presenting: 676 µmol/L (ref 11-51) | Glutamine: 1907 µmol/L (ref 486-670)  Citrulline: 0 µmol/L (ref 10-33)  Arginine: 40 µmol/L (ref 57-97) | Lactate: 2.8 mmol/L (ref 0.5-2.2)  Ornithine: 120 µmol/L (ref 47-97) |  | c.1313dupG  homozygous  p.(T439Hfs*52) | dialysis, sodium phenylacetate and sodium benzoate (250 mg/kg/day), carbamylglutamate (100 mg/kg/day), carnitine (50 mg/kg/day), arginine (200 mg/kg/day), vitamin B12, nutrition without protein (with glucose (10mg/kg/min) and lipids (2 g/kg/day) | carbamylglutamate (50 mg/kg/day) and protein restriction (3.5 mg/kg/day) | normal stature, weight and neurological development at age 6 years | Peoc’h 2020 |
| **97.**  Prenatal  (younger sibling of case 96; cousin of case 98) | Family history of NAGS deficiency | Presenting:  65 µmol/L (ref 11-51) | Glutamine: 776 µmol/L (ref 486-670)  Citrulline: 4 µmol/L (ref 10-33)  Arginine: 34 µmol/L (ref 57-97) |  |  | c.1313dupG  homozygous  p.(T439Hfs*52) | nutrition without protein, with glucose (10 mg/kg/min) and lipids (2 g/kg/day), carbamylglutamate (100 mg/kg/day), arginine (200 mg/kg/day) | Carbamylglutamate (50 mg/kg/day), protein restriction (3.5 g/kg/day) | normal stature, weight and neurological development at age 4 years | Peoc’h 2020 |
| **98.**  Neonatal  (cousin of cases 96 and 97) | family history of infant death from hypotonia and coma | Presenting: 117 µmol/L (ref 11-51) | Glutamine: 1820 µmol/L (ref 486-670)  Citrulline: 1 µmol/L (ref 10-33)  Arginine: 19 µmol/L (ref 57-97) | Glutamate: 53 µmol/L (ref 11-51) |  | c.1313dupG  homozygous  p.(T439Hfs*52) | sodium benzoate (250 mg/kg/day), carbamylglutamate (50 mg/kg/day), arginine (200 mg/kg/day), protein restriction (1 g/day) | carbamylglutatmate (50 mg/kg/day) | normal weight, stature, and neurological development at 16 months | Peoc’h 2020 |

**References:**

Al Kaabi EH, El-Hattab AW (2016) [N-acetylglutamate synthase deficiency: Novel mutation associated with neonatal presentation and literature review of molecular and phenotypic spectra.](https://www-ncbi-nlm-nih-gov.proxy.library.emory.edu/pubmed/27570737) *Mol Genet Metab Rep* 8:94-8.

Alm J, Nordenström A, Hallberg B, von Döbeln U, Häberle J (2004) Neonatal Hyperammonemia Successfully Treated with Carbaglu® in a Neonate with N-Acetyl-Glutamate Syntase Deficiency." *Pediatric Research* 56(3): 465.

Bachmann C, Brandis M, Weissenbarth-Riedel E, Burghard R, Colombo JP (1988) [N-acetylglutamate synthetase deficiency, a second patient.](https://www-ncbi-nlm-nih-gov.proxy.library.emory.edu/pubmed/3139931) *J Inherit Metab Dis* 11(2):191-3.

Bachmann C, Colombo JP, Jaggi K (1982) [N-acetylglutamate synthetase (NAGS) deficiency: diagnosis, clinical observations and treatment.](https://www-ncbi-nlm-nih-gov.proxy.library.emory.edu/pubmed/7164912) *Adv Exp Med Biol* 153:39-45.

Bachmann C, Krähenbühl S, Colombo JP, Schubiger G, Jaggi KH, Tönz O (1981) [N-acetylglutamate synthetase deficiency: a disorder of ammonia detoxication.](https://www-ncbi-nlm-nih-gov.proxy.library.emory.edu/pubmed/7453791) *N Engl J Med* 304(9):543.

Bélanger-Quintana A, Martínez-Pardo M, García MJ, Wermuth B, Torres J, Pallarés E, Ugarte M (2003) [Hyperammonaemia as a cause of psychosis in an adolescent.](https://www-ncbi-nlm-nih-gov.proxy.library.emory.edu/pubmed/12942317) *Eur J Pediatr* 162(11):773-5.

Bijarnia-Mahay S, Häberle J, Jalan AB, Puri RD, Kohli S, Kudalkar K, Rüfenacht V, Gupta D, Maurya D, Verma J, Shigematsu Y, Yamaguchi S, Saxena R, Verma IC (2018) [**Urea** **cycle** **disorders** in **India**: **clinical** **course**, **biochemical** and **genetic** **investigations**, and **prenatal** **testing**.](https://www-ncbi-nlm-nih-gov.proxy.library.emory.edu/pubmed/30285816) *Orphanet J Rare Dis* 1;13(1):174.

Burlina AB, Bachmann C, Wermuth B, Bordugo A, Ferrari V, Colombo JP, Zacchello F (1992) [Partial N-acetylglutamate synthetase deficiency: a new case with uncontrollable movement disorders.](https://www-ncbi-nlm-nih-gov.proxy.library.emory.edu/pubmed/1405478) *J Inherit Metab Dis* 15(3):395-8.

Caldovic L, Morizono H, Daikhin Y, Nissim I, McCarter RJ, Yudkoff M, Tuchman M (2004) [Restoration of ureagenesis in N-acetylglutamate synthase deficiency by N-carbamylglutamate.](https://www-ncbi-nlm-nih-gov.proxy.library.emory.edu/pubmed/15480384) *J Pediatr* 145(4):552-4.

Caldovic L, Morizono H, Panglao MG, Cheng SF, Packman S, Tuchman M (2003) [Null mutations in the N-acetylglutamate synthase gene associated with acute neonatal disease and hyperammonemia.](https://www-ncbi-nlm-nih-gov.proxy.library.emory.edu/pubmed/12594532) *Hum Genet* 112(4):364-8.

Caldovic L, Morizono H, Panglao MG, Lopez GY, Shi D, Summar ML, Tuchman M (2005) [Late onset N-acetylglutamate synthase deficiency caused by hypomorphic alleles.](https://www-ncbi-nlm-nih-gov.proxy.library.emory.edu/pubmed/15714518) *Hum Mutat* 25(3):293-8.

Caldovic L, Morizono H, Tuchman M (2007) [Mutations and polymorphisms in the human N-acetylglutamate synthase (NAGS) gene.](https://www-ncbi-nlm-nih-gov.proxy.library.emory.edu/pubmed/17421020) *Hum Mutat* 28(8):754-9.

Cartagena A, Prasad AN, Rupar CA, Strong M, Tuchman M, Ah Mew N, Prasad C (2013) [Recurrent encephalopathy: NAGS (N-acetylglutamate synthase) deficiency in adults.](https://www-ncbi-nlm-nih-gov.proxy.library.emory.edu/pubmed/23250120) *Can J Neurol Sci* 40(1):3-9.

Cavicchi C, Chilleri C, Fioravanti A, Ferri L, Ripandelli F, Costa C, Calabresi P, Prontera P, Pochiero F, Pasquini E, Funghini S, la Marca G, Donati MA, Morrone A (2018) [Late-Onset N-Acetylglutamate Synthase Deficiency: Report of a Paradigmatic Adult Case Presenting with Headaches and Review of the Literature.](https://www-ncbi-nlm-nih-gov.proxy.library.emory.edu/pubmed/29364180) *Int J Mol Sci.* 2018 Jan 24;19(2).

Colombo JP (1994) [N-acetylglutamate synthetase (NAGS) deficiency.](https://www-ncbi-nlm-nih-gov.proxy.library.emory.edu/pubmed/7741005) *Adv Exp Med Biol* 368:135-43.

Corne C, Fouilhoux A, Aquaviva C, Besson G (2011) First French case of NAGS deﬁciency. 20 years of follow up. *Mol Genet Metab* 120(2):275.

Dammers R, Rubio-Gozalbo ME, Robben SG, Bakker JA, Spaapen LJ, Forget PP (2002) [N-acetyl-glutamate synthetase deficiency or porto-systemic shunt associated encephalopathy?](https://www-ncbi-nlm-nih-gov.proxy.library.emory.edu/pubmed/12162617) *Acta Paediatr* 91(6):729.

Elpeleg ON, Colombo JP, Amir N, Bachmann C, Hurvitz H (1990) [Late-onset form of partial N-acetylglutamate synthetase deficiency.](https://www-ncbi-nlm-nih-gov.proxy.library.emory.edu/pubmed/2373115) *Eur J Pediatr* 149(9):634-6.

Elpeleg O, Shaag A, Ben-Shalom E, Schmid T, Bachmann C (2002) [N-acetylglutamate synthase deficiency and the treatment of hyperammonemic encephalopathy.](https://www-ncbi-nlm-nih-gov.proxy.library.emory.edu/pubmed/12447942) *Ann Neurol* 52(6):845-9.

Forget PP, van Oosterhout M, Bakker JA, Wermuth B, Vles JS, Spaapen LJ (1999) [Partial N-acetyl-glutamate synthetase deficiency masquerading as a valproic acid-induced Reye-like syndrome.](https://www-ncbi-nlm-nih-gov.proxy.library.emory.edu/pubmed/10626533) *Acta Paediatr* 88(12):1409-11.

Gessler P, Buchal P, Schwenk HU, Wermuth B (2010) [Favourable long-term outcome after immediate treatment of neonatal hyperammonemia due to N-acetylglutamate synthase deficiency.](https://www-ncbi-nlm-nih-gov.proxy.library.emory.edu/pubmed/19533169) *Eur J Pediatr* 169(2):197-9.

Grody WW, Chang RJ, Panagiotis NM, Matz D, Cederbaum SD (1994) [Menstrual cycle and gonadal steroid effects on symptomatic hyperammonaemia of urea-cycle-based and idiopathic aetiologies.](https://www-ncbi-nlm-nih-gov.proxy.library.emory.edu/pubmed/7837763) *J Inherit Metab Dis*17(5):566-74.

Guffon N, Schiff M, Cheillan D, Wermuth B, Häberle J, Vianey-Saban C (2005) [Neonatal hyperammonemia: the N-carbamoyl-L-glutamic acid test.](https://www-ncbi-nlm-nih-gov.proxy.library.emory.edu/pubmed/16126063) *J Pediatr.* 147(2):260-2.

Guffon N, Vianey-Saban C, Bourgeois J, Rabier D, Colombo JP, Guibaud P (1995) [A new neonatal case of N-acetylglutamate synthase deficiency treated by carbamylglutamate.](https://www-ncbi-nlm-nih-gov.proxy.library.emory.edu/pubmed/7623444) *J Inherit Metab Dis* 18(1):61-5.

Häberle J, Denecke J, Schmidt E, Koch HG (2003b) [Diagnosis of N-acetylglutamate synthase deficiency by use of cultured fibroblasts and avoidance of nonsense-mediated mRNA decay.](https://www-ncbi-nlm-nih-gov.proxy.library.emory.edu/pubmed/14605506) *J Inherit Metab Dis* 26(6):601-5.

Häberle J, Schmidt E, Pauli S, Kreuder JG, Plecko B, Galler A, Wermuth B, Harms E, Koch HG (2003a) [Mutation analysis in patients with N-acetylglutamate synthase deficiency.](https://www-ncbi-nlm-nih-gov.proxy.library.emory.edu/pubmed/12754705) *Hum Mutat* 21(6):593-7.

Heibel SK, Ah Mew N, Caldovic L, Daikhin Y, Yudkoff M, Tuchman M (2011) [N-carbamylglutamate enhancement of ureagenesis leads to discovery of a novel deleterious mutation in a newly defined enhancer of the NAGS gene and to effective therapy.](https://www-ncbi-nlm-nih-gov.proxy.library.emory.edu/pubmed/21681857) *Hum Mutat* 32(10):1153-60.

Hinnie J, Colombo JP, Wermuth B, Dryburgh FJ (1997) [N-Acetylglutamate synthetase deficiency responding to carbamylglutamate.](https://www-ncbi-nlm-nih-gov.proxy.library.emory.edu/pubmed/9427158) *J Inherit Metab Dis* 20(6):839-40.

Kim JH, Kim YM, Lee BH, Cho JH, Kim GH, Choi JH, Yoo HW (2015) [Short-term efficacy of N-carbamylglutamate in a patient with N-acetylglutamate synthase deficiency.](https://www-ncbi-nlm-nih-gov.proxy.library.emory.edu/pubmed/25787344) *J Hum Genet* 60(7):395-7.

Kiykim E, Zubarioglu T (2014) [Low dose of carglumic acid for treatment of hyperammonemia due to N-acetylglutamate synthase deficiency.](https://www-ncbi-nlm-nih-gov.proxy.library.emory.edu/pubmed/25228620) *Indian Pediatr* 51(9):755-6.

Kuchler G, Rabier D, Poggi-Travert F, Meyer-Gast D, Bardet J, Drouin V, Cadoudal M, Saudubray JM (1996) [Therapeutic use of carbamylglutamate in the case of carbamoyl-phosphate synthetase deficiency.](https://www-ncbi-nlm-nih-gov.proxy.library.emory.edu/pubmed/8739970) *J Inherit Metab Dis*19(2):220-2

McNutt MC, Deberardinis R, Gotway G (2018) Deficiency of N-acetylglutatmate synthase (NAGS) presenting as postpartum hyperammonemia. *Mol Genet Metab* 123:250.

Morris AA, Richmond SW, Oddie SJ, Pourfarzam M, Worthington V, Leonard JV (1998). [N-acetylglutamate synthetase deficiency: favourable experience with carbamylglutamate.](https://www-ncbi-nlm-nih-gov.proxy.library.emory.edu/pubmed/9870213) *J Inherit Metab Dis* 21(8):867-8.

Nordenström A, Halldin M, Hallberg B, Alm J (2007) [A trial with N-carbamylglutamate may not detect all patients with NAGS deficiency and neonatal onset.](https://www-ncbi-nlm-nih-gov.proxy.library.emory.edu/pubmed/17510757) *J Inherit Metab Dis* 30(3):400.

Pandya AL, Koch R, Hommes FA, Williams JC (1991) [N-acetylglutamate synthetase deficiency: clinical and laboratory observations.](https://www-ncbi-nlm-nih-gov.proxy.library.emory.edu/pubmed/1779615) *J Inherit Metab Dis* 14(5):685-90.

Peoc'h K, Damaj L, Pelletier R, Lefèvre C, Dubourg C, Denis MC, Bendavid C, Odent S, Moreau C. (2020) [Early care of *N*-acetyl glutamate synthase (NAGS) deficiency in three infants from an inbred family.](https://www.ncbi.nlm.nih.gov/pubmed/32021803) Mol Genet Metab Rep. 2020 Jan 24;22:100558.

Plecko B, Erwa W, Wermuth B (1998) [Partial N-acetylglutamate synthetase deficiency in a 13-year-old girl: diagnosis and response to treatment with N-carbamylglutamate.](https://www-ncbi-nlm-nih-gov.proxy.library.emory.edu/pubmed/9877039) *Eur J Pediatr* 157(12):996-8.

Reigstad H, Woldseth B, Häberle J (2017) [Normal Neurological Development During Infancy Despite Massive Hyperammonemia in Early Treated NAGS Deficiency.](https://www-ncbi-nlm-nih-gov.proxy.library.emory.edu/pubmed/28275973) *JIMD Rep* 37:45-47.

Sancho-Vaello E, Marco-Marín C, Gougeard N, Fernández-Murga L, Rüfenacht V, Mustedanagic M, Rubio V, Häberle J. (2016) [Understanding N-Acetyl-L-Glutamate Synthase Deficiency: Mutational Spectrum, Impact of Clinical Mutations on Enzyme Functionality, and Structural Considerations.](https://www-ncbi-nlm-nih-gov.proxy.library.emory.edu/pubmed/27037498) *Hum Mutat* 37(7):679-94.

Sayarifard F, Hadipour F, Hadipour Z, Häberle J, Shafeghati Y, Sagheb S, Sarkhail P (2016) The First Iranian Case of N-acetyl-glutamate Synthase (NAGS) Deficiency Treated with N-carbamylglutamate. *Am J Med Case Rep* 4(12):384-388.

Schmidt E, Nuoffer JM, Häberle J, Pauli S, Guffon N, Vianey-Saban C, Wermuth B, Koch HG (2005) [Identification of novel mutations of the human N-acetylglutamate synthase gene and their functional investigation by expression studies.](https://pubmed.ncbi.nlm.nih.gov/15878741/) *Biochim Biophys Acta* 1740(1):54-9.

Schubiger G, Bachmann C, Barben P, Colombo JP, Tönz O, Schüpbach D (1991) [N-acetylglutamate synthetase deficiency: diagnosis, management and follow-up of a rare disorder of ammonia detoxication.](https://www-ncbi-nlm-nih-gov.proxy.library.emory.edu/pubmed/2044610) *Eur J Pediatr* 150(5):353-6.

Takanashi J, Barkovich AJ, Cheng SF, Weisiger K, Zlatunich CO, Mudge C, Rosenthal P, Tuchman M, Packman S (2003) [Brain MR imaging in neonatal hyperammonemic encephalopathy resulting from proximal urea cycle disorders.](https://www-ncbi-nlm-nih-gov.proxy.library.emory.edu/pubmed/12812952) *AJNR Am J Neuroradiol* 24(6):1184-7.

Tuchman M, Caldovic L, Daikhin Y, Horyn O, Nissim I, Nissim I, Korson M, Burton B, Yudkoff M (2008) [N-carbamylglutamate markedly enhances ureagenesis in N-acetylglutamate deficiency and propionic acidemia as measured by isotopic incorporation and blood biomarkers.](https://www-ncbi-nlm-nih-gov.proxy.library.emory.edu/pubmed/18414145) *Pediatr Res* 64(2):213-7.

van de Logt AE, Kluijtmans LA, Huigen MC, Janssen MC (2017) [Hyperammonemia due to Adult-Onset N-Acetylglutamate Synthase Deficiency.](https://www-ncbi-nlm-nih-gov.proxy.library.emory.edu/pubmed/27147233) *JIMD Rep* 31:95-99.

Van Leynseele A, Jansen A, Goyens P, Martens G, Peeters S, Jonckheere A, De Meirleir L (2014) [Early treatment of a child with NAGS deficiency using N-carbamyl glutamate results in a normal neurological outcome.](https://www-ncbi-nlm-nih-gov.proxy.library.emory.edu/pubmed/24233332) *Eur J Pediatr* 173(12):1635-8.

Vockley J, Vockley CM, Lin SP, Tuchman M, Wu TC, Lin CY, Seashore MR (1992) [Normal N-acetylglutamate concentration measured in liver from a new patient with N-acetylglutamate synthetase deficiency: physiologic and biochemical implications.](https://www-ncbi-nlm-nih-gov.proxy.library.emory.edu/pubmed/1562355) *Biochem Med Metab Biol* 47(1):38-46.

Williams M, Burlina A, Rubert L, Polo G, Ruijter GJG, van den Born M, Rüfenacht V, Haskins N, van Zutven LJCM, Tuchman M, Saris JJ, Häberle J, Caldovic L (2018) [N-Acetylglutamate Synthase Deficiency Due to a Recurrent Sequence Variant in the N-acetylglutamate Synthase Enhancer Region.](https://www-ncbi-nlm-nih-gov.proxy.library.emory.edu/pubmed/30337552) *Sci Rep* 18;8(1):15436.

Zimmermann A, Bachmann C, Schubiger G (1985) [Liver pathology in a new congenital disorder of urea synthesis: N-acetylglutamate synthetase deficiency.](https://www-ncbi-nlm-nih-gov.proxy.library.emory.edu/pubmed/3936265) *Virchows Arch A Pathol Anat Histopathol* 408(2-3):259-68.
